# Phospholipid Metabolites in Recurrent Glioblastoma: In Vivo Markers Detect Different Tumor Phenotypes before and under Antiangiogenic Therapy

**DOI:** 10.1371/journal.pone.0056439

**Published:** 2013-03-08

**Authors:** Elke Hattingen, Oliver Bähr, Johannes Rieger, Stella Blasel, Joachim Steinbach, Ulrich Pilatus

**Affiliations:** 1 Institute of Neuroradiology, Goethe-University Hospital Frankfurt, Frankfurt, Germany; 2 Dr. Senckenberg Institute of Neurooncology, Goethe-University Hospital Frankfurt, Frankfurt, Germany; University Hospital of Heidelberg, Germany

## Abstract

**Purpose:**

Metabolic changes upon antiangiogenic therapy of recurrent glioblastomas (rGBMs) may provide new biomarkers for treatment efficacy. Since in vitro models showed that phospholipid membrane metabolism provides specific information on tumor growth we employed in-vivo MR-spectroscopic imaging (MRSI) of human rGBMs before and under bevacizumab (BVZ) to measure concentrations of phosphocholine (PCho), phosphoethanolamine (PEth), glycerophosphocholine (GPC), and glyceroethanolamine (GPE).

**Methods:**

^1^H and ^31^P MRSI was prospectively performed in 32 patients with rGBMs before and under BVZ therapy at 8 weeks intervals until tumor progression. Patients were dichotomized into subjects with long overall survival (OS) (>median OS) and short OS (<median OS) survival time from BVZ-onset. Metabolite concentrations from tumor tissue and their ratios were compared to contralateral normal-appearing tissue (control).

**Results:**

Before BVZ, ^1^H-detectable choline signals (total GPC and PCho) in rGBMs were elevated but significance failed after dichotomizing. For metabolite ratios obtained by ^31^P MRSI, the short-OS group showed higher PCho/GPC (p = 0.004) in rGBMs compared to control tissue before BVZ while PEth/GPE was elevated in rGBMs of both groups (long-OS p = 0.04; short-OS p = 0.003). Under BVZ, PCho/GPC and PEth/GPE in the tumor initially decreased (p = 0.04) but only PCho/GPC re-increased upon tumor progression (p = 0.02). Intriguingly, in normal-appearing tissue an initial PEth/GPE decrease (p = 0.047) was followed by an increase at the time of tumor progression (p = 0.031).

**Conclusion:**

An elevated PCho/GPC ratio in the short-OS group suggests that it is a negative predictive marker for BVZ efficacy. These gliomas may represent a malignant phenotype even growing under anti-VEGF treatment. Elevated PEth/GPE may represent an in-vivo biomarker more sensitive to GBM infiltration than MRI.

## Introduction

Antiangiogenic treatment, one of the most important new therapies for recurrent glioblastomas (rGBMs), is based on the rationale that cessation of perfusion in the highly vascularized GBMs will induce tumor starvation. Usually, the vascular endothelial growth factor A (VEGF-A), which is mediating the strong neoangiogenesis and increased vascular leakage of GBMs is targeted by the humanized monoclonal IgG antibody bevacizumab (BVZ). Treatment of GBMs is typically monitored with MR imaging, assuming contrast enhancement as the major indicator of recurrent tumor growth [1]. BVZ has been shown to induce high response rates, but diminishing neovasculature and vascular leakage upon BVZ per se results in impressive disappearance of contrast enhancement on MRI causing a bias towards overestimation of response rates (‘pseudo-response’) [2], [3]. Thus, the value of contrast enhancing behavior of the tumor for predicting overall survival is still debatable [2], [4], [5]. Newer criteria for assessing disease progression and treatment response in GBM address these problems by including the T2-signal changes [6]. Still, biomarkers should be more specific for monitoring changes in tumor tissue. Advanced MRI techniques can measure specific tissue parameters including concentrations of distinct metabolites with a high potential for being valuable biomarkers [7], [8]. In a previous work which included a patient subgroup from this study, we studied oxygenation and energy metabolism in recurrent GBMs before and after the first cycle of BVZ treatment using quantitative MRI and MR spectroscopic imaging (MRSI) [9]. We found a decrease in tumor oxygenation under BVZ as well as a sustained increase in the low energy metabolite inorganic phosphate and a decrease (compared to normal tissue) in the high energy metabolites adenosine triphosphate (ATP) and phosphocreatine (PCr).

The metabolism of phospholipid cell membrane turnover is one of the major indicators for tumor growth [10]. In many studies on gliomas it has been shown that the choline (Cho) signal detectable by proton (^1^H) MR spectroscopy (MRS) is an important biomarker for tumor cell proliferation and tumor cell density [11]. A recent work demonstrated that the signal intensity ratio of Cho to the metabolic marker for neuronal integrity N-acetyl-aspartat (NAA) changed upon antiangiogenic treatment and that these changes may predict the outcome of therapy [12]. The Cho signal intensity in ^1^H MRSI just measures the integrated concentration of the membrane lipid catabolite glycerophosphocholine (GPC) and the anabolite phosphocholine (PCho). Extensive studies on tumor models in cell culture showed decreased PCho and increased GPC concentrations upon treatment, a modification not visible by the integrated ^1^H Cho signal [13]–[15]. Phosphorus (^31^P) MRSI offers the discrimination between these two compounds and adds their ethanolamine analogues to the detectable parameters, providing a much more comprehensive characterization of lipid membrane metabolism than^ 1^H MRSI [10]. Experimental studies on tumor models suggest increased PCho as marker for malignancy [16]–[18]. Elevated phosphoethanolamine (PEth) was detected in biopsy samples of brain tumors and has been attributed to glioma cells under growth arrest [14], [17].

The large majority of in vivo ^31^P spectroscopic studies of human brain tumors at 1.5 T investigated the phosphomonoester (PME) and the phosphodiester (PDE) region of the spectra without resolving separate resonances [19]–[25]. In contrast, in vivo ^1^H decoupled ^31^P MR spectroscopic studies have been successfully performed at 1.5 T of human brain tumors resolving resonances for PCho, PEth, GPC and glycerophosphoethanolamine (GPE) [26], [27]. However, at magnetic field strengths of 3T and above, in vivo ^1^H decoupled ^31^P MRSI provides a more reliable discrimination and quantification of two PME (PCho and PEth) and two PDE (GPC and GPE) signals [9].

In our previous study we found an increased PEth/GPE ratio in recurrent GBMs before BVZ [9]. This finding encouraged us to initiate an extended prospective study focusing on changes of phospholipid metabolism in recurrent GBMs before and in the follow–up during BVZ treatment. MRSI was performed before and after starting BVZ therapy and in the follow–up until tumor progression. We aimed to evaluate whether the profile of MRS visible lipid metabolites before BVZ gives us predictive markers for the overall survival in patients with progressive GBM under BVZ. For patients with pronounced therapy success, we monitored the impact of BVZ with the aim to identify markers suitable to monitor therapy.

## Materials and Methods

### Study subjects

The study was approved by the local ethics committee of the faculty of medicine at the local University (Ethik-Kommission des Fachbereichs Medizin der Johann Wolfgang Goethe-Universität, reference number 4/09-SIN 01/09), and written informed consent was obtained from each participant prior to inclusion. Spectroscopic examination was part of a prospective registry study performed on a patient cohort receiving BVZ as compassionate treatment [4]. We included 32 consecutive patients (median age 53, range 31–70 years, 11 females and 21 males) with recurrent glioblastomas, 50% of these patients were included in a previous study [9]. Participants underwent an MRI examination before, 8 weeks after the first cycle of BVZ and -in the follow-up- every 8 weeks until a new tumor recurrence was observed.

All patients received bevacizumab at a dose of 10 mg/kg body weight intravenously every other week. For 9 patients irinotecan was co-administered at a dose of 125 mg/m^2^, while the others received BVZ only. Enrollment was restricted to patients with the histological diagnosis of a GBM with radiologically confirmed recurrence according to the updated response assessment criteria (RANO) for high-grade gliomas [1], [6]. The group included 30 primary and 2 secondary GBMs. The recommendation for an individual treatment schedule with bevacizumab was in the responsibility of the treating physician. All patients were pretreated with radiochemotherapy with temozolomide, adjuvant temozolomide, and further chemotherapy before they were referred for bevacizumab therapy. After starting bevacizumab therapy the treatment response, e.g. tumor progress under BZV, was monitored according the RANO criteria [6].

### MR study protocol

MRSI and MRI of the brain were performed on a 3 T whole body system (Magnetom Trio, Siemens Medical AG, Erlangen, Germany) using a double tuned ^1^H/^31^P volume head coil (Rapid Biomedical, Würzburg, Germany). The MRSI protocol was planned on T2-weighted (T2-w) images in three orientations. For ^1^H MRSI a transversal slice (240×240 mm^2^ FOV, 16×16 matrix, circular weighted acquisition scheme with 1 acquisition, 1.5 s repetition time (TR), 30 ms echo-time (TE)) was recorded within a measurement time of 4∶45 min. Visually guided by coronal and sagittal anatomical slices the 2D ^1^H slab was positioned in the middle of the tumor tissue. The volume of interest (VOI), selected by a combination of point resolved selective spectroscopy (PRESS) and outer volume suppression, covered the center of the recurrent tumor area and the contralateral normal brain tissue. For ^31^P MRSI, a 3D MRSI slab (240×240×200 mm^3^ FOV, 8×8×8 matrix, elliptical weighted acquisition scheme with 10 acquisitions at the center of k-space, 2 s repetition time, 2000 ms TR, 60° pulses, 2.3 ms delay between excitation and recording of FID, 10∶44 min measurement time) aligned to the ^1^H MRSI slice was recorded. Before spatial Fourier transformation the matrix size was doubled in all dimensions by zero filling. Within this process, the ^31^P slab was adjusted by grid-shifting to provide an ideal matching of ^31^P and ^1^H voxels, i.e. the ^1^H slice was positioned in the center of a ^31^P slice and the in-plane ^31^P grid matched the ^1^H grid just exhibiting twice the scale.

### Selection of region of interest (ROI)

A graphical user interface developed at our institute was used for image guided selection of the tumor ROI based on the T2-weighted references images and contrast enhanced T1-weighted images recorded at a previous examination (up to 7 days before starting bevacizumab). An experienced radiologist (E.H.) visually selected the voxels of the tumor area, which was defined as solid contrast enhancing tumor mass avoiding necrotic areas. Whenever possible, control tissue was selected from a region contralateral to the tumor position, provided that there was no obvious tumor or edema. Alternatively, another adjacent healthy region of the contralateral hemisphere was selected. In the follow-up examinations under bevacizumab the tumor area frequently showed no or just faint contrast enhancement. In these cases the tumor area was delineated based on signal changes in T2-weighted images according to following features: intermediate signal intensity between grey matter and vasogenic edema; inhomogeneous signal changes; blurred gray-matter junction with altered cortical ribbon; lack of ‘fingers of edema’ [28]. These criteria together with the contrast enhancement provided a collection of clues to differentiate tumor from normal tissue, necrosis and from edema.

For the ^31^P data, a second control area was positioned in the temporo-occipital region of the unaffected hemisphere. For all but two patients this area was sufficiently far apart from the tumor to neglect the signal spreading from adjacent voxels, which was caused by the poor point spread function. Thus, data from regular tissue were provided which were not affected by ROI positioning and therefore were comparable between different patients.

### Data analysis

Data were sampled from voxels within the tumor ROI and the control ROIs. For each target area the ^31^P voxels were selected first, followed by selection of the ^1^H voxel within the ^31^P voxel (i.e. 4 ^1^H voxel per 1 ^31^P voxel). The program jMRUI [29], which performs a fitting routine of the FID in the time domain, was used to analyze the^ 31^P data. The model function was composed of 14 exponentially decaying sinusoids. Six of those had identical damping and were corresponding to peaks assigned to PCr, PEth, PCho, GPC, GPE, and inorganic phosphate (Pi). PCr was adjusted to 0 ppm and constraints for the chemical shifts of the other signals except for Pi were applied as a fixed difference with regard to the position of PCr. ATP was represented by 7 exponentially damped sinusoids, defining each multiplet by the respective number of peaks with identical damping and adequate amplitude ratios. The^ 1^H spectra were analyzed with the software LCModel (Provencher, downloadable test version at: http://s-provencher.com) [30] which performs a frequency domain fitting routine using a linear combination of metabolite specific model spectra. Baseline correction was performed including macromolecules. All ^1^H MRSI spectra from the selected voxels were visually assessed for artifacts according to the criteria described by Kreis et al. [31] to exclude voxels with poor spectra quality. The appearance of a lipid signal was not taken as exclusion criteria since lipids are considered as typical indicator for necrotic regions. In the fit of the ^31^P spectra, all signals were generally assigned correctly leaving a flat line for the residual with equal distribution of noise.

Signal intensities were corrected for T1 and T2 relaxations using values reported previously [32] and averaged over the target region. Concentrations for ^1^H detectable metabolites were then calculated by referring to an independent measurement in a spherical phantom containing an aqueous solution of 100 mmol/l acetate as calibration standard. A TR of 10 s was applied to avoid T1 saturation. For ^31^P data, a spherical phantom with 20 mmol/L phosphate served as calibration standard (TR 60 s).

### Statistical analysis

#### 1. Recurrent GBM against control

All patients with qualified MRS data were included in a survival analysis (Kaplan-Meier). To answer the question whether there are pre-treatment metabolite changes predicting overall survival under bevacizumab, the entire group was split at the median overall survival time resulting in a group with long overall survival (long-OS) and a group with short overall survival (short-OS). Signal intensities and their ratios for specific metabolites were analyzed with analysis of variance (ANOVA) comparing the groups with long-OS with short-OS for tumor and control tissue of the contralateral hemisphere. Differences between tumor tissue and control tissue were tested using ANOVA with repeated measurements. Significance (p < 0.05) was tested by contrast analysis in ANOVA.

#### 2. Follow-up

For patients with a recurrence-free period of at least 2 follow-up MR examinations, changes in tumor metabolites were determined comparing 3 examinations: the examination before BVZ therapy was commenced, the examination after the first cycle of BVZ and the examination revealing tumor progression under BVZ [6]. Longitudinal changes in metabolite concentrations or their ratios were analyzed using ANOVA with repeated measurements. Significance was tested with contrast analysis in ANOVA.

## Results

The median survival time for the entire patient group was 243 days, resulting in 16 patients with OST > 243 days (long-OS) and OST < 243 days (short-OS). For one patient the results regarding energy metabolism deviated considerably from 2 times standard deviation due to an extraordinary high signal of inorganic phosphate. We considered these data as outlier and excluded it from further statistical analysis regarding energy metabolism.

**Figure 1:**
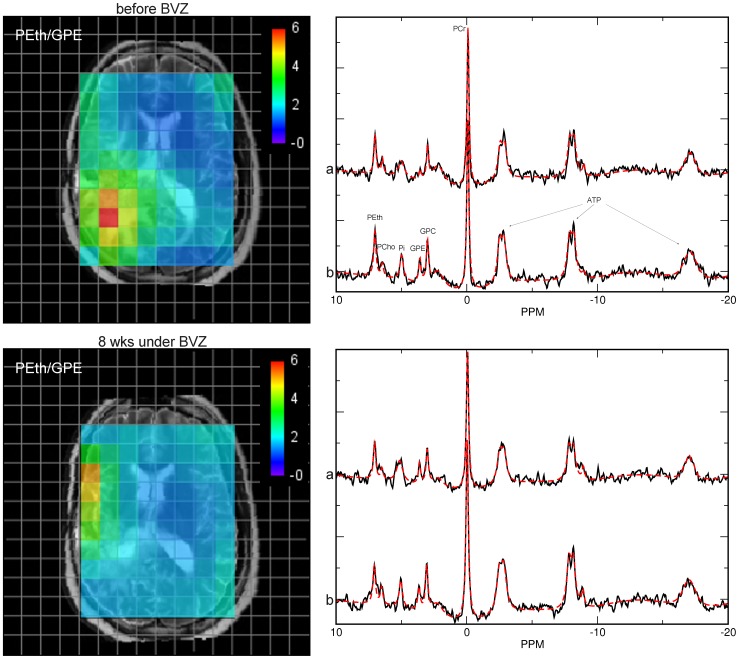
Representative PEth/GPE intensity map from a patient with recurrent GBM in the right parietooccipital lobe overlaid on the T2-weigthed anatomical slice before (upper line) and 8 weeks after starting BVZ therapy (lower line). The color scale specifies the levels of the PEth/GPE ratio of each ^31^P voxel, ranging from red for the highest ratios to blue for the lowest. To avoid unreasonable high values at very low GPE concentrations, the minimum GPE signal intensity was fixed at the accuracy for the data fitting procedure given by the ‘Cramer Rao Lower Bounds’ obtained from the jMRUI results table. Highest PEth/GPE ratios are found in the tumor area (area of high signal on the T2-w slice) of recurrent GBM which was decreased 8 under BZV. Representative ^31^P spectra from the tumor area (a) and from the normal-appearing tissue of the corresponding contralateral hemisphere (b) are shown in the right panels. Note that the PGE signal is almost absent whereas the PEth is prominent. Also note the decreased signal intensities of high-energy phosphates PCr and ATP in the tumor tissue compared to control.

From the 32 patients 14 patients were examined at least three times after start of Bevacizumab treatment until the tumor progressed again. Representative spectra from recurrent GBM and from contralateral control tissue are shown in Fig. 1.

Especially for the tumor area many voxels (about 40%) had to be rejected since they did not meet the quality criteria.

### 1. Membrane lipid metabolites

#### Lipid metabolites in recurrent GBMs before treatment (long-OS and short-OS) compared to control tissue of the contralateral hemisphere (Table 1)

**Figure 2:**
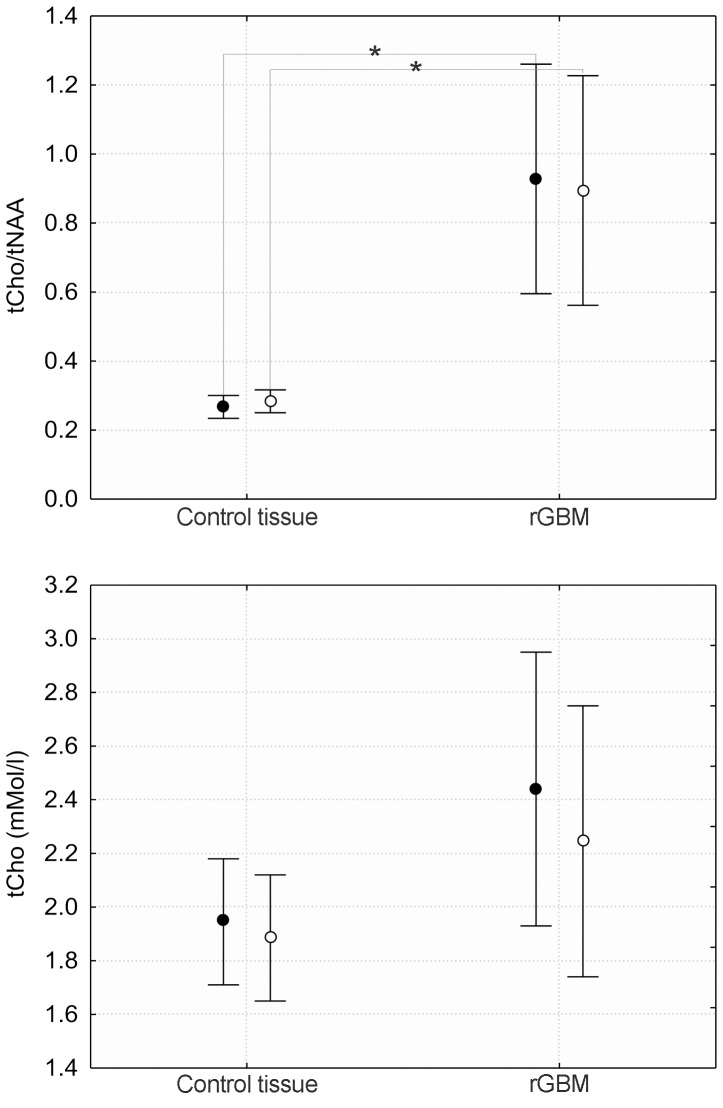
Ratios of tCho/NAA and concentrations of total Cho (tCho) for control and tumor tissue. Bars represent 95% confidence interval according to ANOVA. Closed symbols represent short-OS, open symbols long-OS.

**Figure 3:**
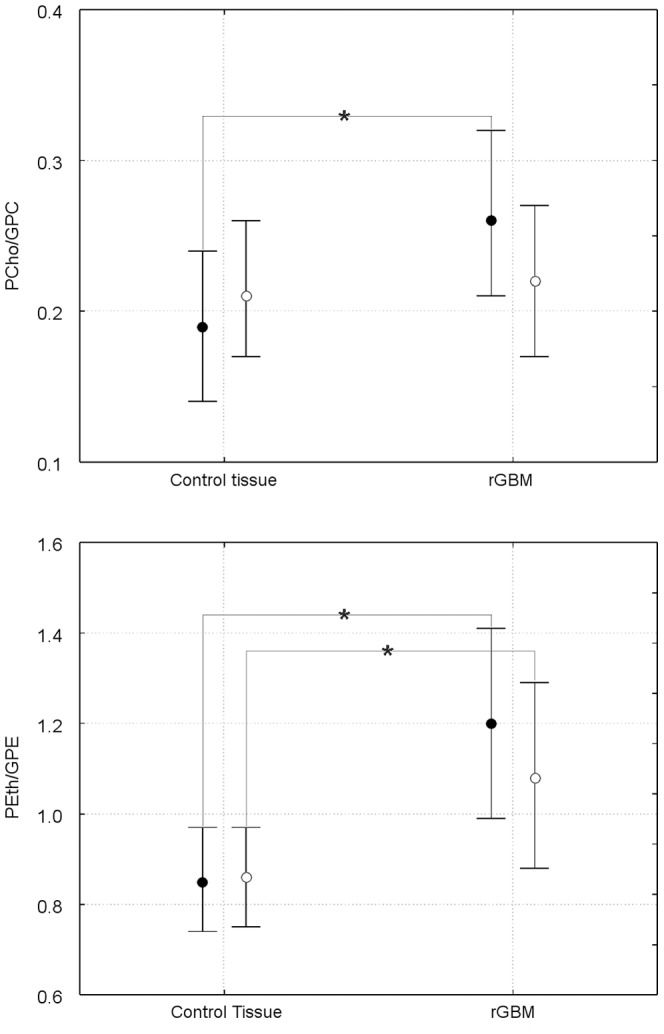
Ratios for ^31^P lipid metabolite concentrations PCho/GPC and PEth/GPE for tumor and control tissue. Closed symbols represent short-OS, open symbols long-OS. * indicates p<0.05 level significance.

a. The concentration of tCho obtained from ^1^H spectra for the entire group was significantly increased (p = 0.04) in tumor tissue. Likely due to diminished statistical power, the significance is lost after separated into long-OS (p = 0.21) and short-OS (p = 0.09) (Fig. 2, lower panel). The tCho/NAA ratios calculated for the tumor and the controls are shown in the upper panel. The pronounced NAA decrease in the tumor was responsible for the highly significant difference between tumor and control for both groups (p<0.001).

b. The PCho/GPC ratio was increased for short-OS (p = 0.004), however, patients from the long-OS group showed no difference of PCho/GPC compared to normal-appearing tissue (Fig. 3). The difference between tumor and control comparing long-OS to short-OS reached a significance level of p = 0.05. There was a significant correlation (R = 0.37, p = 0.04) between the PCho/GPC ratio and survival days.

c. The ^31^P detectable ethanolamine-containing metabolite ratio PEth/GPE for both groups was significantly increased (Fig. 3), long-OS (p = 0.04) and short-OS (p = 0.003).

#### Changes of lipid metabolite upon bevacizumab treatment until repeated tumor recurrence

**Figure 4:**
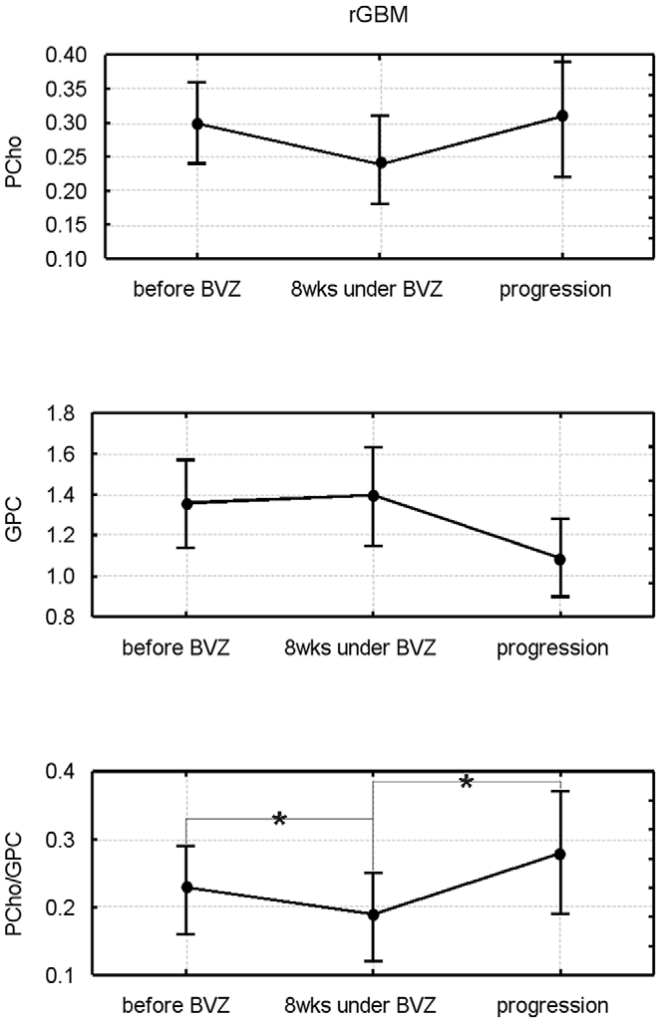
Longitudinal changes in choline-containing metabolites for tumor tissue. The upper rows show concentrations, the lower row the respective concentration ratios. * indicates p<0.05 level significance. The data represent a subgroup with at least 110 days of progression free survival including patients with long-OS and short-OS (please note that 4 patients with long-OS still not had tumor progression).

**Figure 5:**
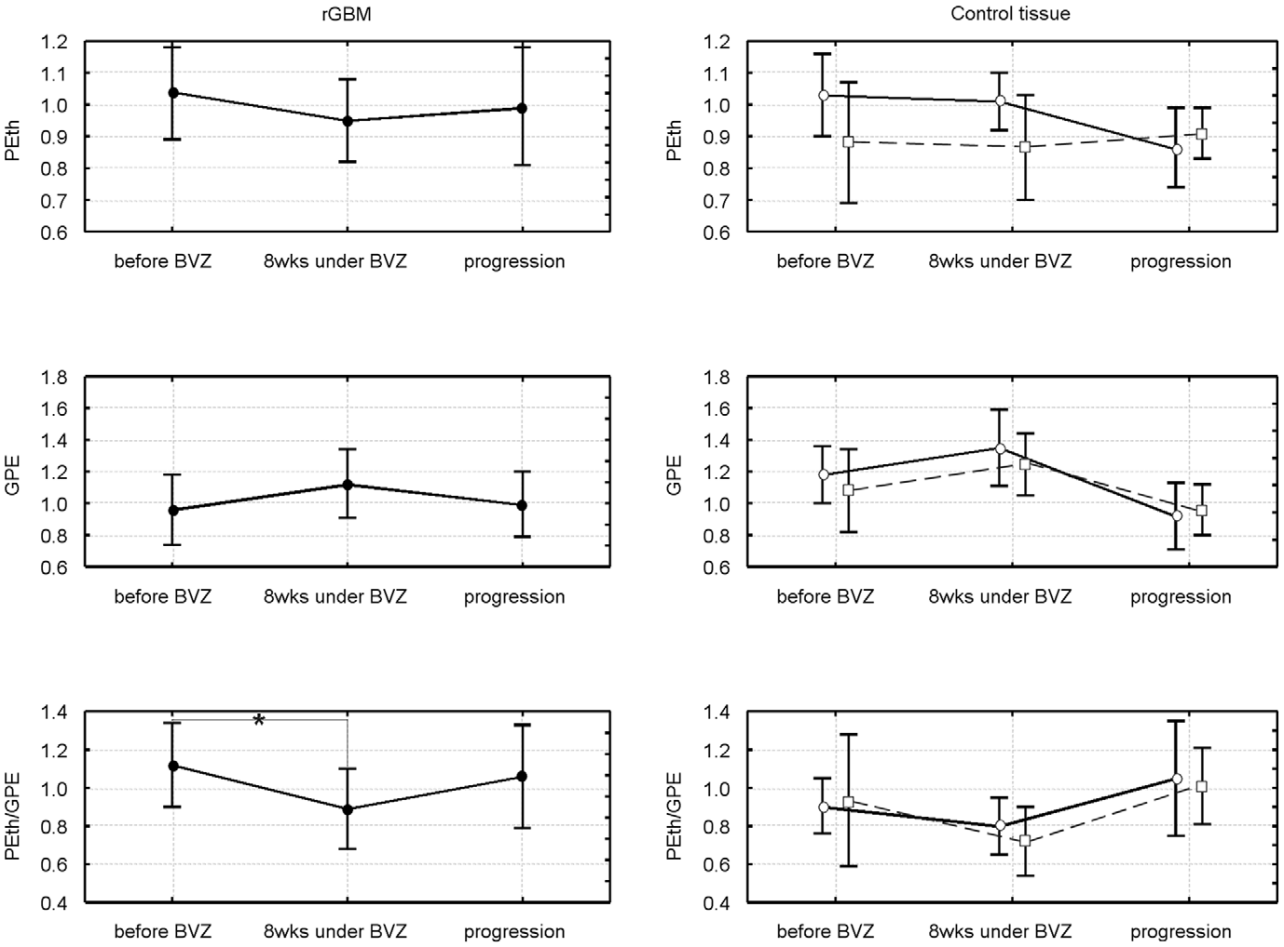
Longitudinal changes in ethanolamine metabolites for tumor and control tissue. The upper rows show concentrations, the lower row the respective concentration ratios. For controls both ROIs (see Material and Methods) are shown with open symbols, the contralateral region with circles and solid lines, the temporal region (depicting ROI in the temporal region independent from tumor position) with squares and dashed lines. The data represent a subgroup with at least 110 days of progression free survival including patients with long-OS and short-OS (please note that 4 patients with long-OS still not had tumor progression).

Neither the ^1^H detectable metabolite concentrations nor their ratios with normal-appearing tissue revealed significant changes after start of bevacizumab compared to the values before treatment (data not shown). Changes in ^31^P metabolites are shown in Fig. 4 for Cho compounds and in Fig. 5 for the Eth compounds. There is a common trend to an increase in the membrane catabolites (GPE, GPC) and a decrease in the anabolites (PEth, PCho) within 8 weeks of treatment, which is reverted when the tumor progresses. However, only the decrease in GPC upon tumor recurrence under BVZ reaches significance (p = 0.007). The ratios of anabolites to catabolites show a significant decrease (p = 0.041 for PEth/GPE, p = 0.039 for PCho/GPC) following treatment, which is reverted upon tumor recurrence (bottom panels of both figures). For the PCho/GPC the re-increase was significant (p = 0.02) exceeding the initial values.

For normal-appearing tissue we found a similar pattern for both control areas with changes almost analogue to the tumor tissue. The initial decrease of PEth/GPE after onset of therapy was significant for the contralateral tissue (p = 0.047) while the increase upon tumor progression was significant for the temporo-occipital region (p = 0.031) (Fig. 5).

### 2. Energy metabolites

Before BVZ, there was a significant difference between tissue of recurrent GBMs and control tissue for PCr/Pi for both groups (p<0.001). It is worth to mention that in the tumor tissue but also in the control tissue the level of PCr/Pi was elevated in patients with long OS compared to the patients with shorter OS (Table 1). A decreased ATP/Pi ratio for tissue of recurrent GBMs was also significant for both, the group of short-OS (p = 0.007) and long-OS (p = 0.03).

**Table 1:** ^1^H and ^31^P metabolites of the tumor site and the control tissue of patients with rGBMs before and 8 weeks after start of BVZ treatment (mean and SD).

Metabolites	Patients with short-OS				Patients with long-OS			
	Before BVZ		Under BVZ		Before BVZ		Under BVZ	
	Tumor	Control	Tumor	Control	Tumor	Control	Tumor	Control
total Cho	2.44 (0.75)	1.95 (0.50)	2.29 (0.64)	2.27 (0.59)	2.31 (1.13)	1.89 (0.39)	2.36 (0.94)	2.13 (0.45)
NAA	**3.10 (1.00)**	**7.97 (2.34)**	*3.19 (1.28)*	*7.51 (2.03)*	**3.21 (1.10)**	**7.32 (1.58)**	*3.56 (1.10)*	*8.58 (1.94)*
total Cr	**4.43 (1.07)**	**6.56 (1.6)**	*4.36 (1.23)*	*6.52 (1.77)*	**4.52 (1.70)**	**6.61 (1.35)**	*4.95 (1.31)*	*7.68 (1.85)*
PCho	**0.35 (0.09)**	**0.29 (0.11)**	0.21 (0.07)	0.26 (0.19)	0.28 (0.09)	0.28 (0.08)	0.27 (0.13)	0.25 (0.13)
PEth	1.11 (0.30)	1.12 (0.28)	0.96 (0.27)	0.99 (0.38)	0.98 (0.19)	0.98 (0.26)	0.93 (0.32)	0.96 (0.22)
GPC	1.39 (0.40)	1.53 (0.38)	1.39 (0.48)	1.39 (0.49)	1.40 (0.51)	1.42 (0.34)	1.44 (0.54)	1.29 (0.32)
GPE	**1.04 (0.50)**	**1.35 (0.38)**	1.07 (0.36)	1.13 (0.44)	1.00 (0.39)	1.15 (0.22)	1.09 (0.31)	1.25 (0.36)
Pi	**1.61 (0.67)**	**1.25 (0.51)**	1.20 (0.38)	1.16 (0.41)	**1.26 (0.40)**	**1.06 (0.30)**	1.18 (0.48)	1.11 (0.34)
PCr	**3.42 (0.74)**	**3.74 (0.79)**	*2.98 (0.82)*	*3.29 (0.95)*	**3.31 (0.68)**	**3.66 (0.70)**	3.22 (1.05)	3.38 (0.72)
ATP	1.95 (0.40)	2.05 (0.49)	1.85 (0.64)	2.04 (0.46)	1.72 (0.57)	1.71 (0.48)	1.46 (0.82)	1.55 (0.72)
PCho/GPC	**0.26 (0.10)**	**0.19 (0.07)**	0.16 (0.07)	0.19 (0.18)	0.22 (0.10)	0.21 (0.10)	0.20 (0.10)	0.20 (0.14)
PEth/GPE	**1.20 (0.45)**	**0.85 (0.16)**	0.95 (0.33)	1.02 (0.86)	**1.08 (0.35)**	**0.86 (0.25)**	0.89 (0.37)	0.82 (0.23)
ATP/Pi	**1.41 (0.55)**	**1.75 (0.42)**	1.63 (0.64)	1.81 (0.59)	**1.43 (0.42)**	**1.67 (0.53)**	1.21 (0.69)	1.47 (0.76)
PCr/Pi	**2.43 (0.90)**	**3.21 (0.73)**	2.61 (0.82)	2.89 (0.81)	**2.82 (0.85)**	**3.61 (0.84)**	2.95 (0.98)	3.15 (0.68)

Patients were separated in two groups: patients with long-term survival-time (OS) and patients with short-term OS referring to the median survival days of the whole patient cohort. Significant differences between tumor and controls are marked in bold (p<0.05) and 8 weeks after onset of BVZ treatment are marked in italic.

**Figure 6:**
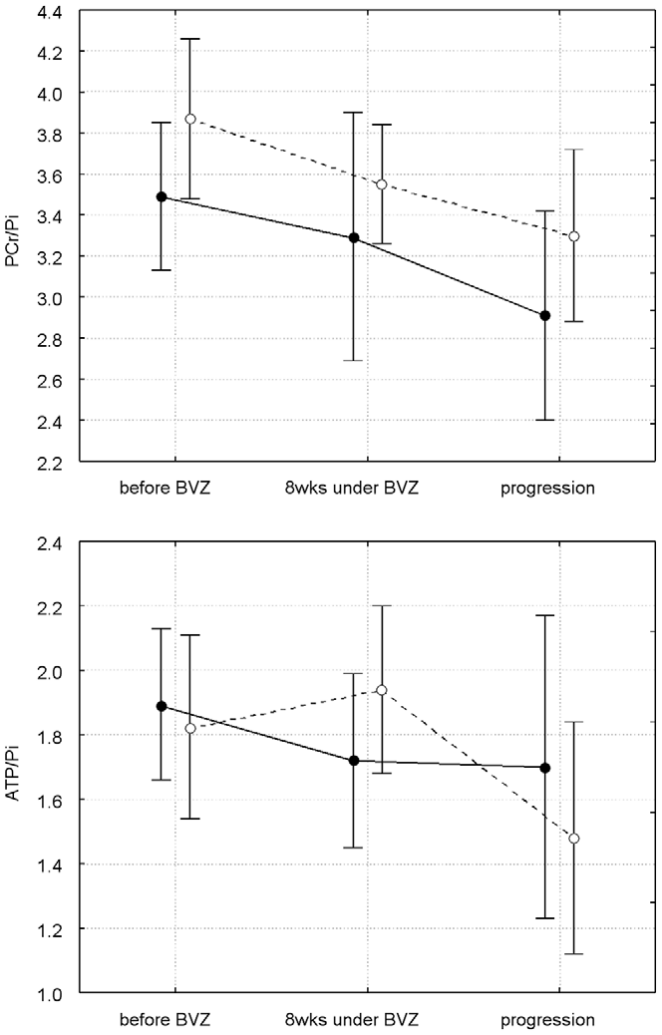
Longitudinal changes of high energy phosphate metabolite concentration. Closed represent tumor, open signals control tissue. Values for the first two time points are different from Table 1, since the data represent a different subgroup (6 patients from the short-OS group, 7 patients from the long-OS group). All patients included in this figure had no confirmed tumor progression at the second time point and could be examined until tumor progression.

For tumor tissue the energy metabolite ratios remained decreased during the whole follow-up period (Fig. 6).

In the normal-appearing control tissue, there was a continuous decrease in the PCr/Pi and ATP/Pi ratio. However, only the ATP/Pi decrease at tumor progression compared to the first post BVZ values was significant (p = 0.034, open symbols and dotted line in lower panel of Fig. 6).

## Discussion

The ^31^P and ^1^H metabolite ratios of membrane phospholipid metabolism and the high energy phosphates (PCr, ATP) were significantly different in recurrent GBMs before BVZ treatment compared to normal-appearing brain tissue of the respective contralateral hemisphere. While ^1^H MRSI could detect differences between normal-appearing tissue the ^31^P data were much more specific: The ratio of PCho/GPC, an indicator for tumor cell proliferation, was increased in patients who survived a shorter period under the following BVZ therapy. In the following we will discuss the pathobiochemical processes underlying the metabolic changes and evaluate the potential of MRSI to monitor antiangiogenic treatment.

Although partial volume effects from necrotic and hemorrhagic tissue may affect the overall voxel concentration, an increased tissue concentration of tCho is typical for malignant GBMs [11]. In this study, a voxel selection rigorously excluding those areas based on anatomical and spectral quality criteria was required to reveal a significant difference. In addition, tCho does not discriminate between lipid anabolites and catabolites relying on the assumption that an increase of one (potentially the anabolic) component is not accompanied by a decrease in the other. Thus, the discrimination between GPC and PCho using ^31^P MRS reflects changes in tumor metabolism with higher accuracy. In vitro studies showed that PCho is the dominant membrane lipid metabolite in proliferating tumor cells and tumor tissues [10], [17]. Formation of PCho by the cholinkinase α is the first step in the synthesis of phosphatidylcholine by the Kennedy pathway [10], [33]. Many malignant tumors including glioma cell lines have been shown to overexpress choline kinase [34], [35]. Several oncogenes increase choline kinase activity when expressed in mouse fibroblasts [33] and hypoxia-inducible factor-1 alpha signaling up regulates choline kinase expression in prostate cancer [36]. While there is a vast amount on information on breast and prostate cancer cell lines [16], [33] little work has been published on gliomas. Gillies at al. [17] found that the PCho content of rat glioma cells decreased during the conversion from the exponential growth to stationary growth phase. Apart from its role as phospholipid membrane precursor, PCho may also act as a second messenger in cell growth signaling [37]. Further the low GPC levels during tumor progression under BVZ (Fig. 5) may indicate increased glycerophosphodiesterase activity which enhances cancer cell migration [38]. Therefore the use of PCho/GPC ratios may improve the diagnostic value due to the opposite concentration changes of these metabolites in tumor tissue.

The presented study is the first in evaluating lipid metabolism of adult brain tumors in vivo by distinguishing the choline- and ethanolamine-containing metabolites. Referring to the in vitro results, we expected increased PCho concentrations and PCho/GPC ratios in recurrent GBMs compared to normal-appearing brain tissue of the respective contralateral hemisphere before BVZ therapy. For the short-OS patients we observed significantly higher PCho/GPC ratios compared to controls. In contrast no significant PCho/GPC increase was found for the long-OS group (Table 1). The difference of tumor PCho/GPC increase was significant when comparing both groups. Further, the pretreatment PCho/GPC ratio correlated with survival days measured from start of BVZ therapy. While the small PCho signal may limit the accuracy of our measurement the results are supported by experimental data emphasizing augmented PCho as a surrogate marker for the malignant potential of gliomas

Phosphatidylethanolamine is another important phospholipid, also called cephalin because it is abundant in the brain and spinal cord [10]. About 45% of brain phospholipids are phosphatidylethanolamines [39]. The Eth-containing pool of membrane lipids is in exchange with for Cho-containing metabolites by the S-adenosyl methionine-mediated sequential N-methylation of phosphatidylethanolamine to synthesize phosphatidylcholine [40]. We found that a significantly increased PEth/GPE ratio in patients with short and long OS serves as the dominant and accurate marker of tumor tissue in recurrent GBMs.

Knowledge of ethanolamine-containing lipid metabolism from human in vivo data is still limited by methodological problems: In ^1^H MR spectra, the in vivo signals of the metabolites PEth and GPE are not distinguishable from the background leaving ^31^P MRS as only resort for measuring ethanolamine membrane metabolites in vivo. Yet, those ^31^P MRS examinations require high fields (≥ 3T) or at least ^1^H decoupling [26], [41]. In a pilot study, measuring ^1^H decoupled ^31^P spectra at 1.5 T, Albers et al. observed elevated PEth/GPE ratios in primitive neuroectodermal tumors [27].

Animal and cell culture studies of experimental in vivo tumors found increased PCho and PEth levels, which were interpreted as elevated rates of membrane synthesis [10], [42]. The extension of this finding to human brain tumors is corroborated by information available from ex vivo studies from biopsy samples. Elevated PEth levels were measured by chromatography analysis of samples from neuroblastomas [43], medulloblastomas and to a lesser extent from GBMs [44], [45]. High-resolution magic angle spinning MRS from low-grade and high-grade glioma samples revealed that concentrations of PEth and PCho increased while GPE and GPC decreased with higher malignancy [46]. All these results suggest that the metabolism of the membrane lipid phosphatidylethanolamine (PtdEth) is linked with metabolism of neuronal cells or their pluripotent precursor cells.

According to in vitro studies [17], [18] PEth increase may be considered as marker of non-proliferating glioma cells, whereas PCho increase indicates the exponential growth phase. Thus, the elevated ratio of PEth/GPE as predominant marker for metabolic change in the presented study hints to non-proliferating cells which would confirm data from literature that the growth fraction of in vivo GBM cells is small [44]. Especially, in case of recurrent GBMs the micro milieu should provide unfavorable conditions for tumor cell proliferation considering the preceding treatment effects like cytotoxicity, secondary gliosis and endothelial damage. In contrast to the in vitro cell studies, our in vivo data revealed that the high PEth/GPE ratio was mainly caused by lower GPE levels. The difference may be attributed to the in vivo tumor conditions which represent a scenario of limited nutrient and oxygene supply while cultured cells usually grow under abundant supply of nutrients. In vivo tumor cells may utilize endogenous resources as described above for the Cho-releasing GPC catabolism [38]. An analogous cleavage of GPE should result in the observed changes for the PEth/GPE ratio.

In summary, our results corroborate the role of PCho as indicator of malignant tumor behavior, whereas the PtdEth metabolism may be attributed to more ‘inactive’ tumor cells with lower membrane lipid turn-over.

### Follow-up on BVZ treatment

While tCho was increased in the initial measurement, we could not detect any significant changes in the first follow up examination. This is in accordance with a previous study by Kim et al. [12] which reported an increase in the tCho concentration in the first phase (up to day 28) of treatment, followed by a decrease (day 56). Kim et al. interpreted the tCho decrease between day 28 and 56 as metabolic changes related to the antitumor effect of the therapy. Our ^31^P MRS data clearly indicate a shift in the concentration from the anabolic component to catabolic component (Figures 4, 5), which corroborates the hypothesis of antitumor activity during this phase of the treatment. Since the tCho signal intensity shows the aggregated concentration of anabolites and catabolites a decrease of one component may be compensated by an increase in the other resulting into no changes for the ^1^H detectable choline signal. As discussed above, PCho/GPC and PEth/GPE are indicators for tumor malignancy and growth and are therefore adequate to monitor effects of targeted antiangiogenic therapy. In the time course of BVZ therapy PCho/GPC increased again when the tumor progressed exceeding even the initial values. Venkatesh et al. showed in vitro that PCho acts as metabolic MR biomarker to monitor GBM treatment effects [47]. The authors found a decrease of PCho in GBM cell cultures treated under inhibition of the rapamycin signaling pathway which is highly activated on GBMs. It is discussed that this pathway increases the transcription of hypoxia inducible factor 1α (HIF-1α) which controls the expression of choline kinase α.

### Energy metabolism

Experimental and in vivo spectroscopic studies of gliomas and other brain tumors showed a variety of changes in the high-energy phosphate metabolism [10]–[17], [48]. A decrease of the PCr signal intensity or the intensity ratio of PCr to the ‘low-energy’ phosphate Pi was the most consistent finding [20], [21], [23], [28], [47], and was assigned to tumor hypoxia caused by inadequate vascularization. Different levels of ATP were found in primary brain tumors [14], [17], [20]. In our study, decreased PCr concentrations as well as the increased Pi concentrations of recurrent GBMs may be assigned to an additional vascular injury from preceding radiation and chemotherapy. Further studies investigating newly-diagnosed GBMs should reveal how secondary tissue changes upon therapy may influence the metabolite concentrations. Upon treatment with BVZ, PCr/Pi and ATP/Pi ratios remained low during the whole follow-up including the period of tumor progression. Therefore, the tumor growth under low-energy conditions implicates that these tumors consist of hypoxia-resistant malignant cells. It is worth to mention that tumor-inducible highly malignant tumor cells are discussed to be more hypoxia-resistant.

### Normal appearing brain tissue

An important finding of this study is that changes of ^31^P metabolites upon treatment were not only detected in recurrent tumor but also within the normal-appearing control tissue of the contralateral hemisphere. Under VEGF treatment we found a significant increase of PEth/GPE in normal-appearing brain areas as the tumor progressed preceded by an initial decrease of this ratio. We can exclude that partial volume effects had caused this phenomenon since the PEth/GPE changes were found even in temporo-occipital areas distant from the tumor. The initial metabolite changes may be the result of general treatment effects. However the re-increase of PEth/GPE in the normal-appearing tissue concomitant with tumor progression cannot easily be explained by treatment effects. Its increase together with the decrease in the ATP/Pi ratio may indicate proliferation of glioma cells. This would support the hypothesis that bevacizumab promotes a diffuse infiltrating, gliomatosis-like phenotype at progression [49]. However, the concept of BVZ-induced gliomatosis could not be confirmed in other conventional MR studies [50], [51]. Herein it should be raised that there is no imaging ‘gold standard’ to detect tumor infiltration and even the FLAIR sequence may fail to show histopathological paths of tumor invasion. FLAIR is influenced by T1- and T2-relaxation times and both may be changed especially under therapy [52]. On the other hand the sensitivity of MRS to detect pathologies in normal appearing brain tissue has been shown for brain tumors [53], [54]. Especially the Cho/NAA index, also known as CNI, turned out to be a very sensitive marker indicating infiltration of glioma cells even outside the area with higher T2-signal [8], [55]–[57].

Despite the above mentioned evidence, a significant and continuing BVZ effect on energy metabolism in normal brain tissue also is still disputable. The ATP/Pi decrease may be caused by unspecific BVZ effect on normal brain tissue. BVZ is the human antibody acting against the vascular endothelial growth factor VEGF, which is highly expressed in glioblastomas but also in other proliferating tissue. Reparative treatment effects like gliosis could induce some VEGF increase which may be antagonized with BVZ.

### Methodological considerations

The visual delineation of tumor areas is challenging in pre-treated rGBMs since no imaging feature may reliably differentiate between vital tumor tissue, therapy related changes or micronecrosis. Especially under antiangiogenic therapy the contrast enhancement may be missed and the tumor area has to be defined on T2-weighted images. Further, tumor borders and tumor edema are often infiltrated by glioma cells. Considering a possible bias of visual tumor delineation, our results strongly suggest that changes of the membrane lipid metabolites may help to detect ‘vital’ tumor tissue under antiangiogenic therapy.

Partial volume effects caused by the rather coarse spatial resolution and signal bleeding between adjacent voxels are a major drawback in ^31^P MRS. Our data show changes in control tissue which were analogue to the tumor metabolite changes. Especially for data from the contralateral side, where vicinity between tumor and control tissue cannot always be avoided, this may indicate bleeding of signal from the tumor voxels. However, the same pattern of longitudinal changes was also observed in the second control area, which was selected far distant from the tumor site, confirming the statement that normal appearing tissue is affected as well. In any case, it is unlikely that the observed metabolite changes demonstrated in this study are just the result of a partial volume effect due to an increase in the proportion of healthy and tumor tissue. Nevertheless, there is an urgent need to improve the spatial resolution. More advanced MR methods which exploit the ^31^P/^1^H J-coupling of choline and ethanolamine compounds involved in membrane metabolism by using heteronuclear polarization transfer, present a promising approach to improve the S/N and spatial localization of ^31^P MRS in the future [58]. Further, robust automated post processing routines should be established to facilitate clinical use of the promising ^31^P MRSI.
